# A Fair Assessment of Evaluation Tools for the Murine Microbead Occlusion Model of Glaucoma

**DOI:** 10.3390/ijms22115633

**Published:** 2021-05-26

**Authors:** Marie Claes, Joana R. F. Santos, Luca Masin, Lien Cools, Benjamin M. Davis, Lutgarde Arckens, Karl Farrow, Lies De Groef, Lieve Moons

**Affiliations:** 1 Department of Biology, Leuven Brain Institute, KU Leuven, 3000 Leuven, Belgium; marie.claes@kuleuven.be (M.C.); Joana.Santos@nerf.be (J.R.F.S.); luca.masin@kuleuven.be (L.M.); lien.cools@kuleuven.be (L.C.); lut.arckens@kuleuven.be (L.A.); Karl.Farrow@nerf.be (K.F.); lies.degroef@kuleuven.be (L.D.G.); 2 Neuro-Electronics Research Flanders, 3001 Leuven, Belgium; 3 Vlaams Instituut voor Biotechnologie (VIB), Center for Brain & Disease Research, 3000 Leuven, Belgium; 4 Imec, 3001 Leuven, Belgium; 5 Institute of Ophthalmology, University College London, London EC1V 9EL, UK; benjamin.davis@stfc.ac.uk; 6 Central Laser Facility, Science and Technologies Facilities Council, UK Research and Innovation, Didcot OX11 0QX, UK

**Keywords:** glaucoma, ocular hypertension, microbead occlusion model, retinal ganglion cells, optical coherence tomography, anterior chamber depth, electroretinography, scotopic threshold response, dendritic retraction, neurodegeneration

## Abstract

Despite being one of the most studied eye diseases, clinical translation of glaucoma research is hampered, at least in part, by the lack of validated preclinical models and readouts. The most popular experimental glaucoma model is the murine microbead occlusion model, yet the observed mild phenotype, mixed success rate, and weak reproducibility urge for an expansion of available readout tools. For this purpose, we evaluated various measures that reflect early onset glaucomatous changes in the murine microbead occlusion model. Anterior chamber depth measurements and scotopic threshold response recordings were identified as an outstanding set of tools to assess the model’s success rate and to chart glaucomatous damage (or neuroprotection in future studies), respectively. Both are easy-to-measure, in vivo tools with a fast acquisition time and high translatability to the clinic and can be used, whenever judged beneficial, in combination with the more conventional measures in present-day glaucoma research (i.e., intraocular pressure measurements and post-mortem histological analyses). Furthermore, we highlighted the use of dendritic arbor analysis as an alternative histological readout for retinal ganglion cell density counts.

## 1. Introduction

As a leading cause of irreversible blindness, glaucoma continues to place a heavy burden on millions of people worldwide. Apart from the inability to restore vision loss, current therapeutic options are incapable of halting disease progression in all glaucoma patients. Despite a myriad of research efforts, novel treatment routes do not reach or pass clinical trial. This inefficient clinical translation can be partially explained by the lack of validated preclinical models and readouts [1].

A multitude of animal species has been used to study the physiopathology of glaucoma [2], yet rodents have become the preferred model organism due to the broad array of available genetic and experimentally induced models and readout approaches, all as recently reviewed by Pang and Clark [1]. In general, genetic and experimentally induced glaucoma models are looked upon as chronic vs. acute, respectively. Chronic genetic models, e.g., the DBA/2J mouse strain, are popular due to a closer resemblance to human progressive pathology. Unfortunately, the asynchronous and variable disease onset, progression, and severity observed in these models often results in constraints regarding experimental set-up in the search towards novel glaucoma therapies, hence explaining the need for experimentally induced models that offer a higher degree of investigational control [1]. The only chronic experimental glaucoma model is the microbead occlusion model. An intracameral injection of microbeads is used to occlude the conventional aqueous humor outflow pathway, thereby provoking a mild and chronic glaucomatous pathology that phenocopies the key glaucomatous features (i.e., elevated intraocular pressure (IOP), retinal ganglion cell (RGC) degeneration, and optic nerve damage), along with a slow disease progression. Yet, the mild phenotype (reports show RGC loss as low as 7–12% at 4 to 12 weeks after glaucoma onset [3,4,5,6,7]) also represents one of its weaknesses, as it leaves a rather small window to chart glaucomatous damage or neuroprotection.

Other significant drawbacks of the microbead model include mixed success rates and weak reproducibility between different experiments and laboratories, despite abundant protocol optimizations (including the need of repeated injections; co-injection of viscoelastic agents; and a wide range of optimized microbead characteristics such as type, size, or concentration). Hence, glaucoma research would benefit from robust readouts to assess the successful induction of the model. In ocular hypertension-dependent glaucoma models such as the microbead occlusion model, IOP elevation is the gold standard to define the success rate. Nonetheless, measuring IOP in mice is challenging and influenced by various factors (e.g., lighting, diurnal fluctuations, anesthesia), often resulting in imprecise and inconsistent data. Given the modest IOP elevation in the microbead occlusion model, the rise in IOP could be obscured by the variable nature of the technique. For this purpose, we explored the use of anterior segment optical coherence tomography (OCT) imaging as an easier-to-measure parameter to evaluate the success rate of ocular hypertension induction in microbead-injected mice.

Confronted with the abovementioned drawbacks of the microbead occlusion model (i.e., mixed success rate, weak reproducibility, and limited window of opportunity for charting RGC neurodegeneration or survival), we aimed to explore whether focusing on readouts for early stage pathological changes could overcome these issues. This focus is especially relevant given the irrevocable nature of glaucomatous neurodegeneration where therapies should interfere as early as possible in the course of the disease. Indeed, the neurodegeneration field (including Alzheimer’s and Parkinson’s diseases) has shifted its research focus towards the presymptomatic disease stages, acknowledging that neuronal loss is preceded by a (often long) phase of neuronal dysfunction and that central nervous system function can only be preserved by tackling these early disease stages. In the glaucoma field, pinpointing the primary cause and the chronological order of subsequent cellular processes is a long-standing debate. Existing research, however, recognizes a differential vulnerability in the sub-compartments of RGCs (i.e., dendrites vs. soma vs. axon vs. synapse) [8]. Previous reports have disclosed that RGC neurites display one of the earliest signs of glaucomatous damage [9,10,11]. Moreover, as dendrites and axons are critical for RGC function, functional deficits presumably precede actual RGC loss [9]. Within the chronic microbead occlusion model, the glaucomatous stress imposed on the RGCs during the first weeks might not (yet) be severe enough to fatally damage them. On the basis of current insights into glaucoma pathogenesis, we evaluated various readouts that enable early stage disease characterization and subsequent therapy intervention by looking at rapid-onset structural (axon and dendrite morphology) and functional (axonal transport and electrophysiology) changes. Our ambition was to select in vivo approaches that allow for longitudinal disease monitoring, together with a high translatability to the clinic, in order to potentially narrow the gap between preclinical and clinical research in glaucoma research.

## 2. Results

### 2.1. Gold Standard Evaluation Tools: Intraocular Pressure (IOP) and Retinal Ganglion Cell (RGC) Loss

**Figure 1:**
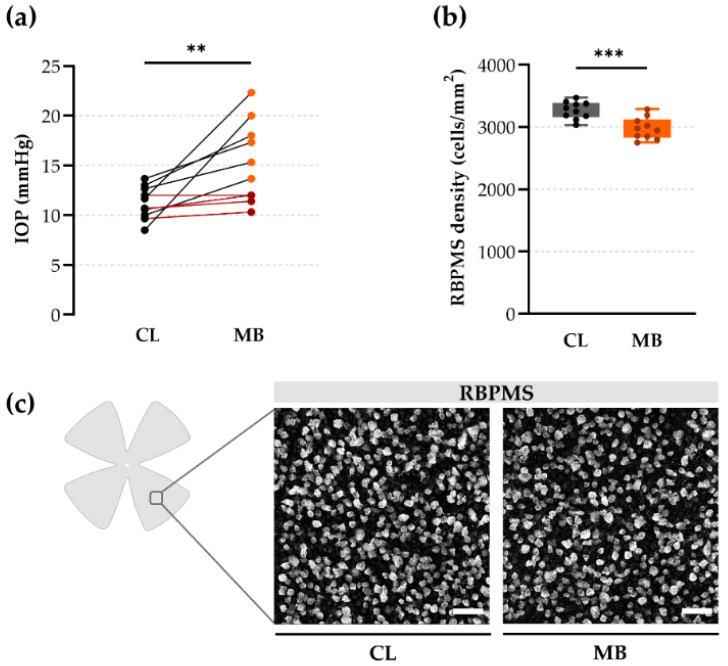
Intracameral microbead injection led to a mild glaucoma phenotype. (**a**) Intraocular pressure (IOP), measured with a rebound tonometer, was increased in microbead-injected eyes at two weeks post-injection, in comparison to the contralateral eyes. Of the 10 mice, 4 did not show elevated IOP (defined as an IOP lower than the mean IOP value of the contralateral eye + one standard deviation) (shown in red). Unpaired two-tailed *t*-test, ** *p* ≤ 0.01, *n* = 10. (**b**) RGC density of entire RBPMS-stained flatmounts was calculated by an automated deep learning tool (RGCode) [13] and revealed a mild RGC loss at five weeks post-injection, as compared to contralateral eyes. Unpaired two-tailed *t*-test, *** *p* ≤ 0.001, *n* = 10. (**c**) Representative photomicrographs of mid-peripheral regions of RBPMS-stained flatmounts revealed no evidently visible changes in RGC densities across contralateral control and microbead-injected eyes. Scale bar = 50 µm. CL = contralateral eyes and MB = microbead-injected eyes.

The protocol of the microbead occlusion model was adapted from Yoko and Belforte et al. [12]. Briefly, 2 µL non-transparent, paramagnetic microbeads (4.5 µm diameter) were injected with a single intracameral injection and re-positioned towards the trabecular meshwork with a handhold magnet. Successful induction of ocular hypertension and glaucomatous damage were evaluated via two old-school parameters: IOP measurements and histological RGC soma counts with the pan-RGC marker RBPMS. The mean IOP was elevated at two weeks post-injection (15.24 ± 1.28 mmHg) as compared to the contralateral controls (11.25 ± 0.52 mmHg; *p* = 0.0097; Hedges’ g = 1.24 with CI_95%_ of 0.47 to 2.04). This mild ocular hypertension led to a moderate RGC loss: the mean RBPMS density of microbead-injected eyes (2978 ± 55 RGCs/mm^2^) was lower than in the contralateral eyes (3269 ± 44 RGC/mm^2^; *p* = 0.0006, Hedges’ g = −1.77 with CI_95%_ of −2.91 to −0.69) at five weeks post-injection (Figure 1b,c). Of note, no difference in RBPMS density was observed between vehicle-injected eyes (3131 ± 41 RGCs/mm^2^) as compared to the contralateral control group (Hedges’ g = −0.98 with CI_95%_ of −2.02 to −0.05), and the contralateral eye was used as reference value during the following experiments. Overall, these data indicated a mild glaucomatous phenotype (22 ± 6% IOP elevation and 9 ± 2% RGC loss) yet left us with a need of more convincing readouts to properly assess glaucomatous damage and create a larger window of opportunity to measure a positive outcome on RGC survival in neuroprotective studies.

### 2.2. Anterior Chamber Depth as an Easy-to-Measure Parameter to Evaluate Model Induction

**Figure 2:**
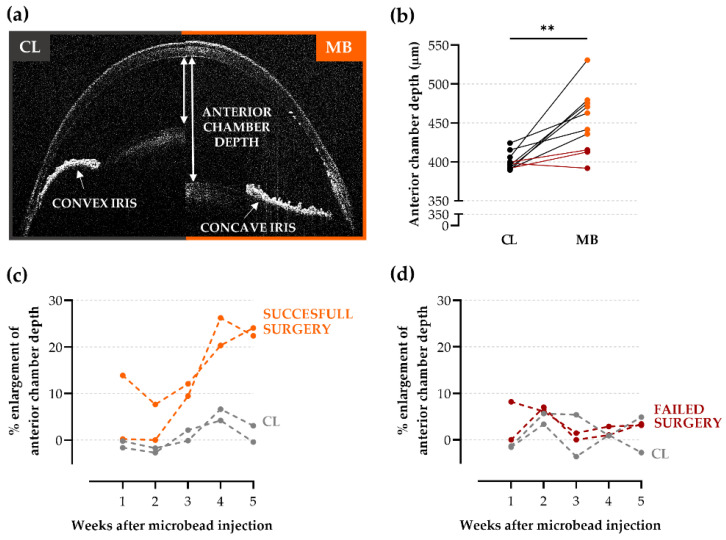
Optical coherence tomography (OCT) of the anterior segment at five weeks after microbead injection. (**a**) Representative anterior segment OCT images of a microbead-injected (right panel, “MB”) and contralateral (left panel, “CL”) eye after pupil dilatation. The measurements of the anterior chamber depth (i.e., the distance between the corneal endothelium and the anterior surface of the lens) are indicated, together with a clear example of a convex iris in a naïve eye vs. a concave, flattened iris after microbead injection. (**b**) Quantification of the anterior chamber depth at five weeks post-injection revealed a substantial enlargement (13 ± 3%) in microbead-injected eyes as compared to the control eyes. Of the 10 mice, 3 did not show elevated anterior chamber depth (defined as anterior chamber depth smaller than the mean anterior chamber depth of the contralateral eye + one standard deviation) (shown in red). Unpaired two-tailed *t*-test, ** *p* ≤ 0.01, *n* = 10. (**c**,**d**) Representative graphs of longitudinal anterior chamber depth follow-up showing the percentage of anterior chamber depth enlargement of mice with a successful (**c**) and failed (**d**) microbead surgery, both in comparison to the contralateral eyes. CL = contralateral eyes and MB = microbead-injected eyes.

Intracameral microbead injection and the resulting ocular hypertension may lead to changes in the structure of the anterior segment that could be useful to evaluate the effectiveness of ocular hypertension induction and serve as a complementary or alternative measure for IOP measurements [14]. OCT imaging of the anterior segment revealed an enlarged anterior chamber depth and more concave, flattened irises after bead injection in comparison to the contralateral controls (Figure 2a). At endpoint measurements (five weeks post-injection), a substantial enlargement of the anterior chamber depth was observed in microbead-injected eyes (452 ± 13 µm) as compared to contralateral controls (400 ± 4 µm; *p* = 0.0012; Hedges’ g = 1.65 with CI_95%_ of 0.71 to 2.62) (Figure 2b). Longitudinal follow-up of anterior chamber depth measurements disclosed baseline values up to and including two weeks post-injection, after which the anterior chamber depth gradually increased (Figure 2c,d). Importantly, a strong, positive correlation (*R*^2^ = 0.84) was found between IOP (two weeks post-injection) and anterior chamber depth (five weeks post-injection) measurements. Hence, anterior chamber depth measurements could provide additional confirmation regarding the success rate of the microbead occlusion model.

### 2.3. Axonal Transport Deficits yet Normal Signal Conduction after Ocular Hypertension

**Figure 3:**
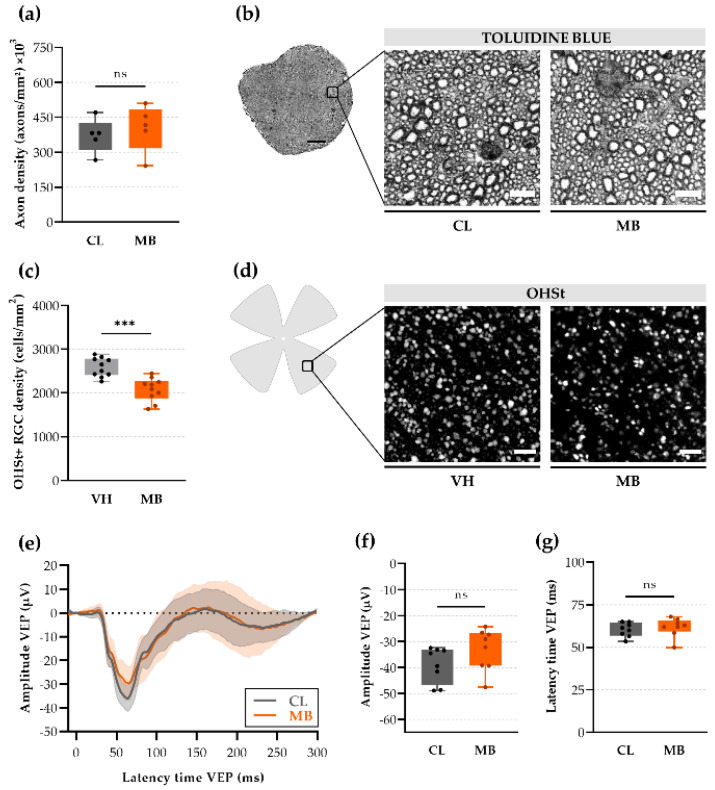
Axonal stress at five weeks after microbead injection. (**a**) Axon density was calculated by an automated deep learning tool (AxoNet) [15] and revealed an unaltered density in the distal optic nerve (3 mm after optic nerve head) upon microbead injection. Unpaired two-tailed *t*-tests, ns = non-significant, *n* = 5. (**b**) Representative images of semi-thin optic nerve cross-sections (contralateral vs. microbead-injected optic nerves) revealed no clear signs of axon degeneration. Scale bar = 5 µm. (**c**) OHSt-labeled (OHSt+) RGC density after retrograde tracing from the superior colliculus was calculated by an automated deep learning tool (RGCode) [13] on entire flatmounts and revealed that axonal transport was notably disrupted in microbead-injected eyes as compared to vehicle-injected controls. Unpaired two-tailed *t*-test, *** *p* ≤ 0.001, n = 10. (**d**) Representative images of mid-peripheral retinal regions after retrograde OHSt tracing revealed a diminished number of OHSt+ RGCs after microbead injection. Scale bar = 50 µm. (**e**) Average (± CI_95%_) full-field flash visually evoked potential (VEP) responses indicated a non-significant but slightly decreased amplitude upon microbead injection as compared to contralateral controls. (**f**,**g**) The amplitude and latency of the VEP response remained unaltered upon microbead injection. Unpaired two-tailed *t*-test, ns = non-significant, *n* = 8. CL = contralateral eyes, VH = vehicle-injected eyes, and MB = microbead-injected eyes.

Multiple studies have shown that RGC axons are among the first RGC sub-compartments to be affected by glaucomatous injury. Hence, we assessed the effects of mild ocular hypertension on axonal integrity at five weeks after microbead injection. In our study, no changes in mean axonal density were observed after microbead injection (404,486 ± 45,063 axons/mm^2^) compared to contralateral optic nerves (372,772 ± 32,556 axons/mm^2^; Hedges’ g = 0.33 with CI_95%_ of −1.13 to 1.86) (Figure 3a,b). Qualitative assessment revealed densely packed axons surrounded by intact myelin sheaths after ocular hypertension, identical to the contralateral controls (Figure 3c). Since no overt axon degeneration was observed at five weeks post-injection, we opted to explore axonal functionality via two studies: (i) evaluating retrograde axonal transport deficits via tracing from the superior colliculus with hydroxystilbamidine metanesulfonate (OHSt) and (ii) assessing anterograde signal propagation via measuring the scotopic full-field visual evoked potential (VEP) at the level of the visual cortex, in response to brief light flashes. The tracing experiment revealed a considerably lower number of OHSt-labeled (OHSt+) RGCs in the microbead-injected eyes as compared to the vehicle-injected controls at six days after tracer introduction (2083 ± 84 vs. 2587 ± 69 OHSt+ cells/mm^2^; *p* = 0.0002; Hedges’ g = −1.99 with CI_95%_ of −2.82 to −1.17) (Figure 3d,f). In contrast, the VEP read-out revealed no attenuation of mean VEP amplitude (−33.1 ± 2.9 vs. −39.0 ± 2.4 µV, glaucomatous vs. contralateral visual cortex, respectively; Hedges’ g = 0.74 with CI_95%_ of −0.44 to 1.77) (Figure 3g–i), nor latency (61.8 ± 2.0 vs. 60.3 ± 1.5 ms, respectively; Hedges’ g = 0.28 with CI_95%_ of −0.86 to 1.34).

### 2.4. Rapid-Onset Changes in Dendritic Arborization after Microbead Injection

**Figure 4:**
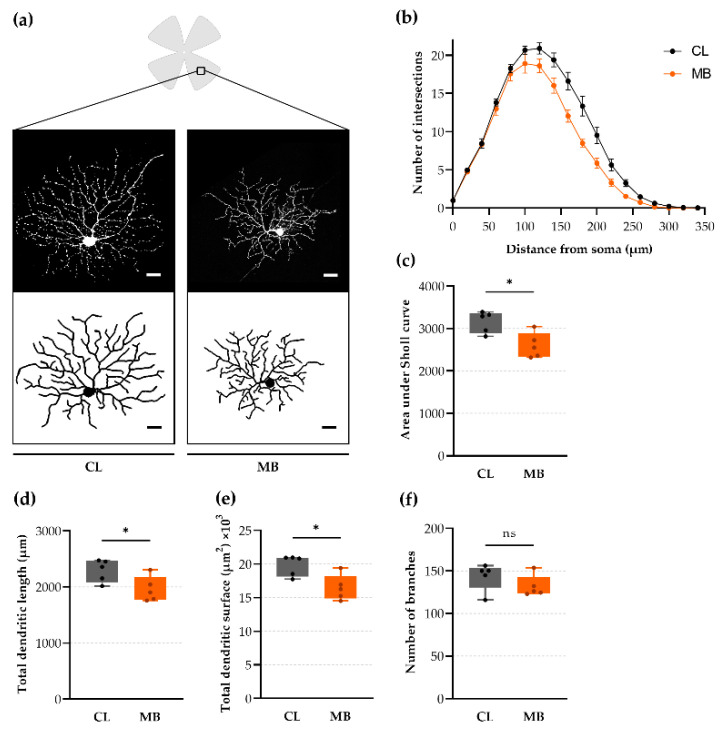
Dendritic architecture was affected by microbead injection. (**a**) Z-stack projection of αRGCs (SMI32-immunopositive) from a Thy1-YFP-H sparsely labeled mouse in the peripheral retina, together with the corresponding en-face view of the dendritic tree, traced with Matlab’s TREES toolbox. Scale bar = 20 µm. (**b**,**c**) Average (± SEM) Sholl profiles (**b**) (i.e., the number of dendrites intersecting concentric circles with a radius increment of 20 µm) and area under the Sholl curve (**c**) revealed dendritic retraction upon microbead injection. (**d**–**f**) Dendritic arbor analysis revealed a markedly lower dendritic length (**d**) and tree surface (**c**), whilst a trend towards a lower number of branches was observed (**e**). Unpaired two-tailed *t*-tests, ns = non-significant, * *p* ≤ 0.05, *n* = 5. CL = contralateral eyes and MB = microbead-injected eyes.

Along with axonal transport deficits, dendritic arbors are amongst the earliest responders to RGC damage in glaucoma models [16,17]. To allow for imaging and 3D reconstruction of individual dendritic arbors, we used transgenic Thy1-YFP-H mice, wherein RGCs are sparsely labeled (1–3%; YFP) [18]. RGCs that showed co-labeling with SMI32 (i.e., αRGCs [19]) were selected for evaluation of dendritic integrity five weeks after microbead injection (Figure 4a). Sholl analysis revealed a substantial reduction of branching complexity from 100 until 300 µm radii from the RGC soma (Figure 4b), resulting in a substantially lower area under the curve (Sholl profile) of microbead-injected eyes *vs.* contralateral controls (2600 ± 133 vs. 3156 ± 113 respectively; *p* = 0.0129; Hedges’ g = −1.82 with CI_95%_of −3.54 to −0.25) (Figure 4c). Microbead injection resulted in dendritic retraction: a smaller mean total length of the dendrites (1959 ± 100 vs. 2291 ± 89 µm, microbead-injected vs. contralateral retinas, respectively; *p* = 0.0380; Hedges’ g = −1.42 with CI_95%_ of −3.11 to 0.23) (Figure 4a,d) and a lower mean surface of the dendritic tree (16,476 ± 843 vs. 19,804 ± 690 µm^2^, respectively; *p* = 0.0157; Hedges’ g = −1.74 with CI_95%_ of −3.61 to −0.16) (Figure 4a,e) was observed. The number of branches did not differ upon microbead injection (132 ± 6 vs. 143 ± 7, respectively; Hedges’ g = −0.72 with CI_95%_of −4.99 to 0.79) (Figure 4a,f).

**Figure 5:**
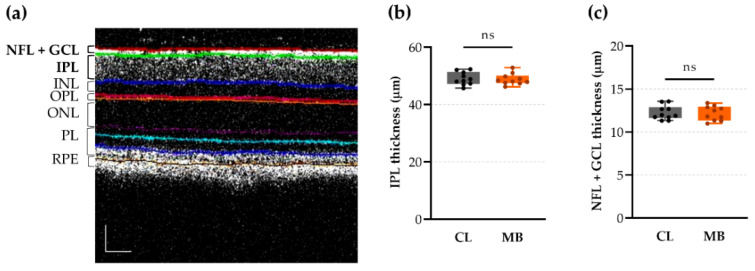
In vivo quantification of retinal layer thickness via optical coherence tomography (OCT) revealed no changes at five weeks after microbead injection. (**a**) Representative OCT image with colored lines indicating the borders of different retinal layers. Scale bar = 50 µm. (**b**) The inner plexiform layer (IPL) thickness was unchanged upon microbead injection. Unpaired two-tailed *t*-test, ns = non-significant, *n* = 10. (**c**) Similarly, the thickness of the combined nerve fiber and ganglion cell layers (NFL + GCL) remained unaltered after microbead injection. Unpaired two-tailed *t*-test, ns = non-significant, *n* = 10. CL = contralateral eyes and MB = microbead-injected eyes.

We next interrogated the thickness of the inner plexiform layer (IPL) via OCT imaging to evaluate dendritic retraction via an in vivo tool (Figure 5a). Unfortunately, quantification of the mean IPL thickness revealed no thinning after microbead injection compared to the contralateral eyes: 48.82 ± 0.62 vs. 49.06 ± 0.71 µm, respectively (Hedges’ g = −0.11 with CI_95%_% of −1.04 to 0.78) (Figure 5b). We also quantified the combined thickness of the nerve fiber and ganglion cell layer (NFL + GCL) to interrogate RGC axonal and soma loss, yet no shrinkage was detected (12.18 ± 0.27 µm vs. 12.24 ± 0.26 µm in microbead-injected and contralateral control eyes, respectively; Hedges’ g = −0.07 with CI_95%_% of −0.93 to 0.91) (Figure 5c).

### 2.5. Changes in Retinal Ganglion Cell (RGC) Electrophysiology as a Precursor for RGC Degeneration

**Figure 6:**
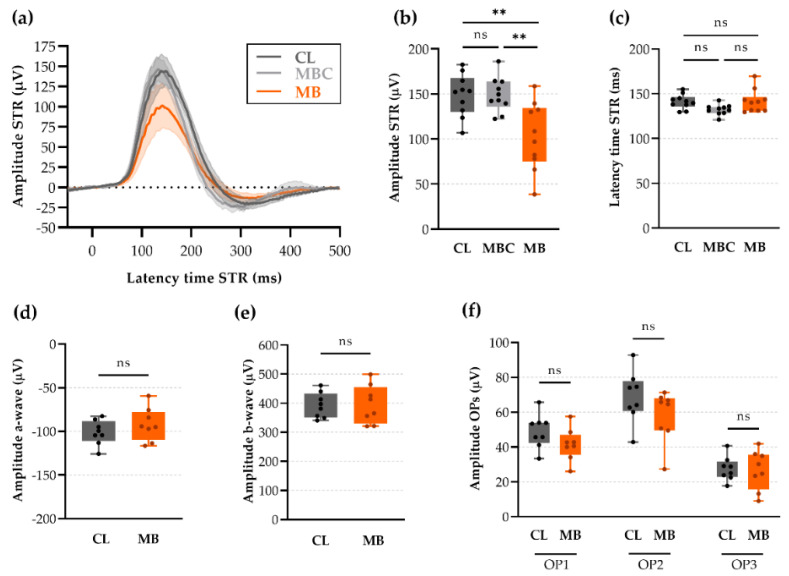
Scotopic threshold response (STR) and full-field flash electroretinogram (ERG) recordings five weeks after microbead injection. (**a**) Average (± CI_95%_) STRs showed a decreased amplitude upon microbead injection. (**b**,**c**) Bar graphs showing a decreased mean STR amplitude and unaltered mean STR latency time in microbead-injected eyes vs. contralateral and microbead-injected controls. One-way ANOVA test with Tukey’s post-hoc tests, ns = non-significant, ** *p* ≤ 0.01, *n* = 10. (**d**–**f**) Bar graphs showing components of the full-field flash ERG, generated primarily by photoreceptor (a-wave, (**d**)), Müller/ON-bipolar (b-wave, (**e**)), and amacrine (oscillatory potentials, OPs, (**f**)) cells, revealing unaltered responses of other retinal cells. Unpaired two-tailed *t*-tests (**d**,**e**) or one-way ANOVA test with Tukey’s post-hoc tests (**f**), ns = non-significant, *n* = 8. CL = contralateral eyes, MBC = microbead-injected control eyes without microbead repositioning towards trabecular meshwork, and MB = microbead-injected eyes.

To further explore rapid-onset glaucomatous changes, we extended our study with scotopic threshold response (STR) recordings, a noninvasive electrophysiology readout for selective interrogation of RGC functionality. Interestingly, five weeks following microbead injection, the STR amplitude was prominently reduced in microbead-injected eyes as compared to their contralateral eyes (103 ± 12 vs. 149 ± 7 µV, respectively; *p* = 0.0031; Hedges’ g = −1.39 with CI_95%_ of −2.29 to −0.44) (Figure 6a,c), whilst the latency time remained unaltered (142 ± 4 vs. 141 ± 3 ms, respectively; Hedges’ g = 0.05 with CI_95%_ of −0.91 to −0.94) (Figure 6b,c). To avoid having the non-transparent, paramagnetic microbeads as a confounding factor, we implemented two control studies: (i) the addition of a microbead-injected control in which an equivalent concentration of microbeads was injected, without relocating the magnetic microbeads towards the iridocorneal angle and hence without inducing ocular hypertension, and (ii) the evaluation of scotopic full-field flash ERGs after microbead injection. Microbead-injected control eyes did not show a decreased STR amplitude (149 ± 6 µV; Hedges’ g = 0.02 with CI_95%_ of −0.90 to −0.90) (Figure 6a,c) or latency time (132 ± 2 ms; Hedges’ g = −1.21 with CI_95%_ of −2.05 to −0.25) (Figure 6b,c) compared to the microbead-injected eyes. Amplitudes and latency times of the a- and b-waves of the full-field flash ERG, reflecting the functionality of other retinal cell types (primarily photoreceptors and Müller/ON-bipolar cells, respectively) that are typically not affected in glaucomatous models, were found unaltered at five weeks after ocular hypertension induction as compared to contralateral controls (Figure 6d,e and Figure S1, Table S1). Likewise, the amplitude and latency time of the oscillatory potentials (OPs), which primarily mirror amacrine cell functioning, remained unchanged at five weeks after microbead injection (Figure 6f and Figure S1, Table S1).

### 2.6. Anterior Chamber Depth and Scotopic Threshold Response (STR) as Easy, Alternative Measures to Assess Model Induction and Disease Progression In Vivo

Integrating the results described above, we conclude that the most outstanding alternatives to the conventional RGC soma counts are the post-mortem evaluation of dendritic arbor changes and in vivo STR recordings of RGC functionality. Second, anterior chamber depth measurements via in vivo OCT imaging represent an easier, less variable tool to assess successful induction of the model in comparison to IOP recordings. Analysis of three independent studies confirmed the reproducibility and low experiment-to-experiment variability of these readouts (Figure S2a,b). The standard deviation and effect sizes of the anterior chamber depth (SD = 12, 13, and 14 µm; Hedges’ g = 1.37, 1.55, and 1.96) and STR measures (SD = 11, 8, and 12 µV; Hedges’ g = −0.72, −1.12, and −1.21) were comparable in the three experiments. One-way ANOVA tests revealed no differences between the anterior chamber depth or STR measurements of different experiments.

Next, the predictive value of the newly proposed and conventional readouts was assessed. Logistic regression with a single input variable revealed a positive association between disease status and IOP or anterior chamber depth, whereas RBPMS+ RGC counts and STR were found to be negatively related with disease status. In other words, the higher the IOP and anterior chamber depth measurements, the more likely it is that model induction was successful, while lower RBPMS+ RGC counts and STR values indicate a higher likelihood of glaucomatous neurodegeneration (Table 1). Moreover, IOP, anterior chamber depth, and STR recordings were all found to be significant predictors of disease state (i.e., normal or glaucomatous eye), yet RBBPMS+ RGC counts were not (Table 1). Multilogistic regression analysis including both STR recordings and RBPMS+ RGC counts suggested no added value of RBPMS+ RGC counts alongside STR measurements (Table 2). Hence, these analyses reinforced the use of anterior chamber depth as an equivalent predictor of model induction to IOP measurements and evidenced that STR measures are preferred over RBPMS+ RGC counts to evaluate glaucomatous damage.

## 3. Discussion

Historically, glaucoma has been classified as an optic neuropathy, with the optic nerve as the initial site of damage. In our study, axonal density counts were unaltered and optic nerve histology looked normal. These observations are in line with several reports in literature, establishing that axonal loss is only visible in advanced disease stages [20]. In fact, various studies have shown that axonal and RGC malfunctioning are typically detected prior to the occurrence of axonal loss [20]. Indeed, retrograde tracing from the superior colliculus revealed clear transport deficits in our study: 20% less RGC somas were filled up with tracer at eight weeks after microbead injection. The biggest drawback of this approach, however, is the need of invasive surgical procedures to introduce the tracer either at the optic nerve or in the superior colliculus. Alternative approaches to document abnormal conduction within post-retinal visual pathways are ex vivo compound action potential recordings in the optic nerve [21] or in vivo VEP recordings from the visual cortex [22,23]. In our study, full-field VEP measurements showed a trend towards attenuation of the VEP signal upon ocular hypertension, yet did not reach statistical significance, perhaps due to limitations in detection. As full-field VEPs are known to lack sensitivity and display highly variable outcomes, it is possible that minor differences in conductivity were not picked up in our study. Akopian et al. [24], however, found reduced VEP amplitudes in their microbead occlusion model, albeit looking at a different endpoint (eight weeks post-injection) and using a protocol with repeated injections that led to a higher RGC loss (≈36%) [24,25]. To probe for subtle, early changes, it is advisable to opt for pattern VEP recordings [22]. Yet, although more sensitive, pattern VEP recordings come with a high installation cost and are not standard lab equipment. In summary, studying axonal dysfunction or degeneration at five weeks after ocular hypertension via either histological analysis of axon density, retrograde tracing, or VEP recordings does not meet our predefined goal: finding an accessible and preferably noninvasive in vivo tool to evaluate early glaucomatous pathology in the microbead occlusion model. Nevertheless, measuring axonal dysfunction is a valuable option for post-mortem evaluations, and VEP recordings may be a suitable in vivo measure at later disease stages.

There is a consensus that glaucoma manifests as early synaptic alterations within the retina, as reviewed by Agostinone and Di Polo [17]. Dendritic retraction is believed to be one of the earliest responses of the RGCs to stress, and can be detected prior to axonal loss [17,26,27,28]. Multiple studies show dendritic changes of αRGCs and other RGC types in acute experimental [29,30,31,32,33,34,35] as well in genetic [36] murine glaucoma models. Likewise, our observations in the chronic microbead model disclosed an aberrant dendritic retraction of αRGCs and a lower area under the Sholl curve at five weeks after microbead injection. These observations are in accordance with the study of El-Danaf and Huberman [37], who showed a shift towards lower intersections in the Sholl plot, as well as a considerably lower dendritic length and field area in murine transient OFF αRGCs as early as one week after microbead injection, as well as the work of Bhandari et al. [38], who revealed a slight reduction in Sholl crossings and a decrease in total dendritic length at five weeks after microbead injection in ON αRGCs of microbead-injected mice. Additionally, Della Santina et al. [26] showed a reduction in dendritic area, length, and number, together with a change in arborization pattern in a Sholl analysis at four weeks after microbead injection in OFF-transient αRGCs. Hence, in our study and in accordance with literature reports, changes in dendritic arbor architecture are early signs of glaucoma. The downside, however, is the substantial time investment that is required for such a study, most often resulting in small sample sizes that reduce the power of the study. Moreover, to avoid overlapping dendritic trees, one requires the use of a transgenic mouse line or a viral vector approach, again imposing limitations on study design. Of note, the sparsely labeled Thy1-YFP-H mice used in this study show a labeling bias for αRGCs [18]. The evaluation of glaucomatous stress on different RGC subtypes would thus demand the use of distinct transgenic mouse lines and/or the use of the few identified molecular markers for RGC subtypes [17]. An alternative, fast, and in vivo readout for dendritic retraction is the evaluation of IPL thinning on OCT images. Unfortunately, no IPL thinning was observed in our study, probably due to the lack of sensitivity of the technique. This observation contrasts the findings of Bhandari et al. [38], who reported an increase in the thickness of the ganglion cell complex (i.e., the three innermost layers of the retina: NFL + GCL + IPL) at five weeks after bilateral microbead injection, presumably as a result of inhibited axonal transport and concurrent axonal swelling. A more sensitive alternative to measure IPL thickness is morphometric analysis on transverse retinal sections, although the advantage of in vivo measurements is lost again. To conclude, if the abovementioned drawbacks of dendritic arbor analysis do not interfere with the envisioned experimental setup, we recommend dendritic arbor analysis as a substitute histological readout for the traditional RGC soma labeling and counts.

From all measures assessed in this study, STR recording ticks all the boxes (i.e., an early onset, easy accessible, and in vivo parameter) and is, in our opinion, the best readout for early stage glaucomatous damage in the microbead occlusion model. At five weeks after microbead injection, the amplitude of the STR peak was reduced by 31%. This observation is in line with the observations of Akopian et al. [25], who observed a 34% decline in STR amplitude at four weeks after single microbead injection. The alterations in STR amplitude in the microbead model are thus of a higher order of magnitude compared to the loss of RBPMS+ RGCs (here 9%) and potentially offer a larger window for therapeutic intervention in neuroprotection studies.

Being one of the major risk factors and currently still the only modifiable factor for glaucoma, elevated IOP typifies the majority of human glaucoma cases. Detecting modest changes in IOP, as seen in the chronic microbead occlusion model, is challenging given the variable nature of the tonometry technique in rodents, to say the least. We opted to explore the use of anterior segment OCT imaging as an easier-to-measure parameter to evaluate the model’s success rate. Upon occlusion of the trabecular meshwork by the microbeads, the conventional outflow of aqueous humor is obstructed and the resulting fluid build-up in the anterior chamber presumably leads to iris flattening, altered iridocorneal angles, and an anterior chamber depth increment. While quantitative assessment of the iridocorneal angle in mice is unfeasible with standard lab equipment, including OCT, the anterior chamber depth is easy measurable. Substantial enlargement of the anterior chamber depth was noted from three weeks after microbead injection in our study, and anterior chamber depth measurements at five weeks after microbead injection showed a positive correlation with IOP values (measured at two weeks post-injection). These findings are in line with the spontaneous increase in anterior chamber depth in aging DBA/2J mice, a genetic glaucoma model [39]. Yet, this is the first report introducing the use of anterior chamber depth measurements as an alternative tool to evaluate the effectiveness of the microbead model.

We proved that changes in anterior chamber depth and STR are significantly altered at five weeks after ocular hypertension and reproducible in independent experiments. Logistic regression analysis was performed to judge the use of these measures as evaluation tools. This analysis revealed that both IOP and anterior chamber depth are good predictors of the success rate of the microbead occlusion model, yet the acquisition of the anterior chamber depth is easier and more robust. Moreover, STR recording, but not RBPMS+ RGC counts, is a significant predictor of disease status (i.e., normal or glaucomatous eyes) at five weeks after induction of the model, and has the added value of being noninvasive and highly translational. Of note, both anterior chamber depth and STR measurements necessitate corneal transparency. As such, we wish to stress the importance of correct microbead positioning and we recommend the use of paramagnetic microbeads that can be manually re-positioned around the circumference of the anterior chamber, as proposed by Ito and Belforte et al. [12]. During the intracameral injection, contact between the microbeads and the inner surface of the cornea should be avoided, as the microbeads have the tendency to perpetually stick to the cornea and could disable OCT and STR measurements [12]. This, together with the avoidance of inflammation after injection, requires an experienced operator. Another remark regarding this study is the lack of determination of an exact quantitative threshold for anterior chamber depth enlargement and STR decline, which was only defined as being higher/lower than the mean of the contralateral eyes ± one standard deviation. We recommend that each laboratory defines their own threshold, tailored to their experimental design and equipment. However, future work could include the development of a linear regression model that predicts STR values for a given IOP or anterior chamber depth measurement. This model would allow for an evaluation of the efficacy of neuroprotective treatments by assessing whether the STR is higher than the predicted value.

## 4. Materials and Methods

### 4.1. Experimental Animals

C57BL/6 or sparsely labeled Thy1-YFP-H (dendritic analysis) mice of either sex were housed under standard laboratory conditions. Experiments started when the mice were between 8 and 12 weeks old. All experimental procedures were approved by the Institutional Ethical Committee of KU Leuven (P007/2018) and were in accordance with the European Communities Council directive of 22 September 2010 (2010/63/EU).

### 4.2. Microbead Occlusion Model

For the intracameral injections, mice were anesthetized with isoflurane (4% for induction, 1.5% for maintenance, Iso-Vet 1000 mg/g, Dechra, Northwich, UK), along with topical eye drops (0.4% oxybuprocaine hydrochloride, Unicaïne, Théa Pharma, Wetteren, Belgium). After pupil dilation (1% tropicamide, Mydriacyl, Novartis Pharma, Basel, Switzerland), 2 µL of 4.5 µm paramagnetic microbeads (Dynabeads™ M-450 Epoxy, ThermoFisher Scientific, Waltham, MA, USA), dissolved in sterile balanced salt solution (BSS plus, Alcon, Fort Worth, TX, USA), was unilaterally injected in the anterior chamber of the right eye, as previously described [13]. Intracameral injection was performed with in-house-made, beveled glass capillaries (1.0/0.75 mm OD/ID with filament, World Precision Instruments, Sarasota, FL, USA) and a MicroSyringe Pump Controller (100 nl per second, Micro4, World Precision Instruments). After injection, the microbeads were re-positioned towards the iridocorneal angle via a handheld magnet. Afterwards, an antibiotic ointment was applied to both eyes (0.3% tobramyxine, Tobrex, Alcon). As vehicle control, an equivalent volume of BSS was injected in the right eye, whilst the microbead-injected control refers to an identical microbead injection without the re-positioning of the microbeads towards the trabecular meshwork. The contralateral eye was always left untouched. Enucleations and optic nerve dissection were performed at five weeks post-injection.

### 4.3. Intraocular Pressure (IOP) Measurements

Using a calibrated rebound tonometer (TONOLAB, type TV02 for rat/mouse measurements, iCare, Vantaa, Finland), we measured the IOP two weeks after microbead injection in awake mice by gently grabbing the fur in the neck whilst still allowing free movement of the animal. The tip of the probe was kept at a distance of 1–3 mm from the center of the cornea. IOP measurements were performed in the morning between 9:00 and 11:00 a.m. under similar lightning settings to minimize the effect of diurnal IOP fluctuations, and were always recorded by the same experienced operator. Six single measurements were recorded, whereafter the four middle measurements were averaged. The same procedure was repeated three times, and the final IOP value was defined as an average of the three previous averages. The contralateral eye was used as an untreated control.

### 4.4. Retrograde Tracing

Unilateral retrograde tracing from the superior colliculus was performed as described by Nadal-Nicolás et al. [40]. Mice were anesthetized by an intraperitoneal injection of a mixture of 75 mg/kg body weight ketamine (Anesketin; Eurovet, Bladel, The Netherlands) and 1 mg/kg medetomidine (Domitor; Pfizer, New York City, NY, USA). The mouse head was fixed in a stereotaxic frame and a 2 × 2 mm^2^ cranial window was made above the superior colliculus. The visual cortex was aspirated, and a gelatin sponge soaked in 0.9% saline containing 4% hydroxystilbamidine metanesulfonate (OHSt) and 10% dimethylsulfoxide (Life Technologies, Carlsbad, CA, USA) was applied on the left superior colliculus. The craniotomy was sealed with elastomer (Kwik-cast, World Precision Instruments), and the skin was sutured. After the procedure, mice were given meloxicam (5 mg/kg, Metacam, Boehringer-Ingelheim, Ingelheim am Rhein, Germany) for post-operative analgesia. Anesthesia was reversed with an intraperitoneal injection of 1 mg/kg atipamezole hydrochloride (Antisedan, Pfizer). A lubricant (Vidisic, Bausch + Lomb, Bridgewater, NJ, USA) was used to prevent dry eyes. Mice were sacrificed six days after tracing. Given the unilateral tracing, we used retinas of different, vehicle-injected mice as controls.

### 4.5. Spectral Domain Optical Coherence Tomography (OCT)

Upon general anesthesia with ketamine/medetomidine (cfr. above) and pupil dilation (0.5% tropicamide, Tropicol, Théa), two different scans (retina and anterior chamber segment) were acquired via spectral domain optical coherence tomography (OCT, Envisu R2210, Bioptigen, Morrisville, NC, USA). Both scans consisted out of 100 consecutive B-scans, each composed of 1000 A-scans, imaged in a rectangular 1.4 × 1.4 mm^2^ (retina) or 3.0 × 3.0 mm^2^ (anterior segment) field. During the imaging, a lubricant (GenTeal, Alcon) was used to improve tear film optics and to hydrate the cornea. Retinal layer thickness was measured using InVivoVue Diver 3.0.8 software (Bioptigen) at 16 locations equally spaced around the optic nerve head and averaged per mouse. Anterior chamber depth was defined as the distance between the corneal endothelium and the anterior surface of the lens, which was calculated using Fiji (ImageJ, version 1.52p) [15] on five, 150 µm spaced B-scans per mouse, positioned around the horizontal plane in the middle of the eye.

### 4.6. Electroretinography (ERG)

Prior to the electroretinogram recordings (Celeris, Diagnosys, Lowell, MA, USA), mice were dark-adapted overnight. Mice were anesthetized with ketamine/medetomidine (cfr. above), and pupils were dilated (0.5% tropicamide, Tropicol, Théa, and 15% phenylephrine hydrochloride, Phenylephrine, Théa) under dim red light. In both protocols—flash electroretinogram (ERG)/visual evoked potential (VEP) vs. scotopic threshold response (STR)—the eyes were alternately stimulated under fully dark-adapted conditions. To measure the ERG and VEP response, we used three active electrodes: two lens electrodes (with integrated touch stimulators) and one needle scalp electrode. The latter was inserted subdermally, above the midline near the visual cortex. The ground and reference electrode were placed in the tail base and cheek, respectively. Full field VEP/ERG responses were recorded at a single flash intensity of 0.05 cd*s/m^2^, averaging 300 brief flashes with an inter-sweep delay of 690 ms. To measure the STR, we solely used two lens electrodes, and the electrode on the contralateral eye was used as a reference. Full field STRs were averaged from 50 white light (6500 K) flashes with a single-flash intensity of 0.0001 cd·s/m^2^ and an inter-sweep delay of 1 s under fully dark-adapted conditions. Espion v6.59.9 software (Diagnosys) was used to define the amplitudes and latency times of each response. The amplitudes and latencies of the ERG components were computed as previously described [41]. VEP and STR amplitudes were defined as the amplitude from the baseline to the trough of the negative VEP response, and from the baseline to the peak of the positive STR. Latencies were determined from flash onset to minimal and maximal amplitudes of the VEPs and STRs, respectively.

### 4.7. Tissue Sampling and Processing

Animals were euthanized by an overdose of sodium pentobarbital (60 mg/kg, Dolethal, Vetoquinol, Aartselaar, Belgium) and transcardial perfusion with 0.9% saline followed by 4% paraformaldehyde (PFA) in phosphate-buffered saline (PBS). Eyes and optic nerves were harvested. Eyes were fixated for one hour in 4% PFA at room temperature and flatmounted after 3 rinsing steps in PBS. Retinal flatmounts were fixated once more for one hour in 4% PFA. The optic nerves were fixated overnight in cold 2% glutaraldehyde buffered at pH 7.3 with 0.05 M sodium cacodylate and 0.05 M saccharose and post-fixated in cold 2% osmium tetroxide in the same buffer. Next, they were dehydrated in a graded acetone series and embedded in Araldite. A series of semi-thin sections (1 µm) was made with a EM UC6 ultramicrotome (Leica, Wetzlar Germany) at 3 mm distance from the optic nerve head, as axon degeneration is believed to progress in a distal-to-proximal manner [42].

### 4.8. Analysis of Axonal Integrity on Semi-Thin Sections

The semi-thin sections were stained with 1% toluidine blue (Sigma-Aldrich, Saint Louis, MO, USA) and 2% sodium borohydrite (Sigma-Aldrich) in distilled water for three minutes at 60 °C. After rinsing with distilled water, they were mounted with Depex (VWR, Radnor, PA, USA). Brightfield microscopy (100 × oil objective, DM6, Leica) was used to image the optic nerves. Axon density was calculated on three distal (3 mm post optic nerve head) cross-sections per optic nerve with the Fiji (ImageJ, version 1.52p) [43] plug-in AxoNet [15], which automatically outlines the optic nerve boundary, counts the RGC axons within this outline, and calculates the corresponding axon density (axons/mm^2^).

### 4.9. Retinal Ganglion Cell (RGC) Soma Counts

Retinal flatmounts were immunostained for the pan-RGC marker RNA-binding protein with multiple splicing (RBPMS). Following a 15 min freezing step in PBS with 0.5% Triton X-100 (VWR) at −80 °C, the flatmounts were incubated overnight with primary rabbit anti-RBPMS antibody (1/250, PhosphoSolutions, Aurora, CO, USA) in PBS with 2% Triton X-100 and 2% pre-immune donkey serum at room temperature. Next, a 2-h incubation with Alexa-647-conjugated donkey anti-rabbit IgG (1/500, Life Technologies) in PBS with 2% Triton X-100 was performed. After rinsing with PBS, the flatmounts were mounted with the anti-fading mounting medium Mowiol (10%, Sigma-Aldrich). RBPMS-immunostained and OHSt-labeled flatmount retinas were imaged with a wide-field epifluorescence microscope (20 × objective, DM6, Leica). RGC densities (RBPMS+ and OHSt+ cells/mm^2^) were calculated with the validated analysis method RGCode, which automatically identifies RBPMS+ or OHSt+ RGCs and retinal boundaries on entire flatmounts and calculates the corresponding RGC density [18].

### 4.10. Retinal Ganglion Cell (RGC) Dendritic Analysis

To assess dendritic changes after microbead injection, we analyzed RGCs in retinas of sparsely labeled Thy1-YFP-H mice, as described by Reinhardt and Li et al. [44]. Briefly, retinal flatmounts were immunostained for green fluorescent protein (GFP, 1:500, Invitrogen) and neurofilament H (NF-H, SMI32, 1:1000, Biolegend, San Diego, CA, USA) for six days at room temperature. High resolution (1024 × 1024 pixels) Z-stacks of the retinal flatmounts were acquired using confocal microscopy (60 × oil objective, 50 µm pinhole aperture, 0.3 µm step size, FV1000, Olympus, Tokyo Japan). Per retina, 15 to 27 SMI32-positive αRGCs with non-overlapping dendritic trees were imaged and analyzed with the TREES Toolbox by Hermann Cuntz et al. [45] in MATLAB (version R2019b, Mathworks, Natick, MA, USA).

### 4.11. Statistical Analysis

Eyes with inflammation or cataract were excluded from analysis. Logistic regression was performed in R (version 4.0.0), after scaling and centering the data automatically with the scale function [46]. All logistic models were exponentiated to report odds ratios with corresponding 95% confidence intervals. Effect size calculations (Hedges’ g) were performed with DABEST [47]. Other calculations (e.g., sample mean and deviation) and statistical tests were performed using Prism 9.0.2 (GraphPad Software, version 9.0.2). Normality was tested with Shapiro–Wilk tests, while other statistical tests were specified in the figure legends, along with the sample size. All data are described as mean ± SEM in the text, whilst figures show box plots with the inter-quartile range (box) and total range (whiskers) of data, unless otherwise indicated. Statistical significance was set as *p* < 0.05 for all analyses.

## 5. Conclusions

We introduced the use of anterior chamber depth measurements via OCT imaging and STR recordings as easy-to-measure, in vivo tools to probe model induction and disease progression during the initial stages of the glaucomatous pathophysiology in the microbead occlusion model. First, the ease of use and fast acquisition time make anterior chamber depth measurements an attractive alternative compared to IOP readings that suffer from high intra-animal, inter-animal, and inter-observer variability, making this measure suitable for routine use in diverse studies. Second, measuring RGC functionality via STR is a better predictor for glaucomatous damage than quantification of RBPMS-immunopositive RGCs. Moreover, STR recordings allow for early detection of glaucomatous damage and yields effects that are large and consistent enough to be rescued by neuroprotectants. The noninvasive STR recordings can be assessed longitudinally during disease progression and can thus allow for frequent and long-term assessment of therapeutic efficacy. Given that OCT and ERG are common methods of early diagnosis of glaucoma in patients [2,48], the use of these tools might, at least in part, help to bridge the gap between preclinical and clinical glaucoma research. Finally, if feasible and opportune, we suggest using dendritic arbor analysis as a more sensitive alternative to RGC density counts in order to assess early stage glaucomatous damage in more in-depth experimental research.

## Figures and Tables

**Table 1:** Simple linear regression to evaluate predictors of glaucomatous disease state, based on different readouts of ocular hypertension and retinal ganglion cell (RGC) degeneration. Intraocular pressure (IOP), anterior chamber depth, and scotopic threshold response (STR) measurements can be employed individually to assess the odds of successful model induction at early disease stages. RBPMS-immunopositive (RBPMS+) RGC counts are not predictors of disease stage.

Variable	Odds Ratio	CI_95%_	*p*-Value
IOP	4.97	1.87–18.87	0.006
Anterior chamber depth	37.64	4.82–1462.22	0.009
RBPMS+ RGC counts	0.48	0.19–1.01	0.076
STR	0.41	0.19–0.78	0.010

**Table 2:** Multilogistic regression comparing the impact of RBPMS-immunopositive (RBPMS+) retinal ganglion cell (RGC) counts and scotopic threshold response (STR) on disease state. Combining both measures suggests no added value of including RBPMS+ RGC counts with STR recordings.

Variable	Odds Ratio	CI_95%_	*p*-Value
RBPMS+ RGC counts	0.52	0.19–1.16	0.141
STR	0.35	0.12–0.80	0.024

## Data Availability

The datasets generated and analyzed in the current study are available from the corresponding author upon reasonable request.

## Supplementary Materials

The following are available online at https://www.mdpi.com/article/10.3390/ijms22115633/s1.

## References

[1] Pang I.-H., Clark A.F. (2020). Inducible rodent models of glaucoma. Prog. Retin. Eye Res..

[2] Evangelho K., Mastronardi C.A., De-la-Torre A. (2019). Experimental Models of Glaucoma: A Powerful Translational Tool for the Future Development of New Therapies for Glaucoma in Humans—A Review of the Literature. Medicina.

[3] Cone F.E., Gelman S.E., Son J.L., Pease M.E., Quigley H.A. (2010). Differential susceptibility to experimental glaucoma among 3 mouse strains using bead and viscoelastic injection. Exp. Eye Res..

[4] Frankfort B.J., Khan A.K., Tse D.Y., Chung I., Pang J.-J., Yang Z., Gross R.L., Wu S.M. (2013). Elevated Intraocular Pressure Causes Inner Retinal Dysfunction Before Cell Loss in a Mouse Model of Experimental Glaucoma. Investig. Opthalmol. Vis. Sci..

[5] Wang R., Seifert P., Jakobs T.C. (2017). Astrocytes in the Optic Nerve Head of Glaucomatous Mice Display a Characteristic Reactive Phenotype. Investig. Opthalmol. Vis. Sci..

[6] Van der Heijden M.E., Shah P., Cowan C.S., Yang Z., Wu S.M., Frankfort B.J. (2016). Effects of Chronic and Acute Intraocular Pressure Elevation on Scotopic and Photopic Contrast Sensitivity in Mice. Investig. Opthalmol. Vis. Sci..

[7] Mukai R., Park D.H., Okunuki Y., Hasegawa E., Klokman G., Kim C.B., Krishnan A., Gregory-Ksander M., Husain D., Miller J.W. (2019). Mouse model of ocular hypertension with retinal ganglion cell degeneration. PLoS ONE.

[8] Della Santina L., Ou Y. (2017). Who’s lost first? Susceptibility of retinal ganglion cell types in experimental glaucoma. Exp. Eye Res..

[9] Morquette J.B., Di Polo A. (2008). Dendritic and synaptic protection: Is it enough to save the retinal ganglion cell body and axon?. J. Neuro-Ophthalmol..

[10] Whitmore A.V., Libby R.T., John S.W.M. (2005). Glaucoma: Thinking in new ways—A rôle for autonomous axonal self-destruction and other compartmentalised processes?. Prog. Retin. Eye Res..

[11] Almasieh M., Wilson A.M., Morquette B., Cueva Vargas J.L., Di Polo A. (2012). The molecular basis of retinal ganglion cell death in glaucoma. Prog. Retin. Eye Res..

[12] Ito Y.A., Belforte N., Cueva Vargas J.L., Di Polo A. (2016). A Magnetic Microbead Occlusion Model to Induce Ocular Hypertension-Dependent Glaucoma in Mice. J. Vis. Exp..

[13] Masin L., Claes M., Bergmans S., Cools L., Andries L., Davis B.M., Moons L., De Groef L. (2021). A novel retinal ganglion cell quantification tool based on deep learning. Sci. Rep..

[14] Yazici A.T., Bozkurt E., Alagoz C., Alagoz N., Pekel G., Kaya V., Yilmaz O.F. (2010). Central Corneal Thickness, Anterior Chamber Depth, and Pupil Diameter Measurements Using Visante OCT, Orbscan, and Pentacam. J. Refract. Surg..

[15] Ritch M.D., Hannon B.G., Read A.T., Feola A.J., Cull G.A., Reynaud J., Morrison J.C., Burgoyne C.F., Pardue M.T., Ethier C.R. (2020). AxoNet: A deep learning-based tool to count retinal ganglion cell axons. Sci. Rep..

[16] Chong R.S., Martin K.R. (2014). Retinal ganglion cell dendrites and glaucoma: A case of missing the wood for the trees?. Expert Rev. Ophthalmol..

[17] Agostinone J., Di Polo A. (2015). Retinal ganglion cell dendrite pathology and synapse loss: Implications for glaucoma. Prog. Brain Res..

[18] Bray E.R., Noga M., Thakor K., Wang Y., Lemmon V.P., Park K.K., Tsoulfas P. (2017). 3D Visualization of Individual Regenerating Retinal Ganglion Cell Axons Reveals Surprisingly Complex Growth Paths. Eneuro.

[19] Krieger B., Qiao M., Rousso D.L., Sanes J.R., Meister M. (2017). Four alpha ganglion cell types in mouse retina: Function, structure, and molecular signatures. PLoS ONE.

[20] Smith M.A., Plyler E.S., Dengler-Crish C.M., Meier J., Crish S.D. (2018). Nodes of Ranvier in Glaucoma. Neuroscience.

[21] Bastian C., Brunet S., Baltan S. (2020). Ex Vivo Studies of Optic Nerve Axon Electrophysiology. Methods in Molecular Biology.

[22] Holder G.E. (2004). Electrophysiological assessment of optic nerve disease. Eye.

[23] Ridder W.H., Nusinowitz S. (2006). The visual evoked potential in the mouse—Origins and response characteristics. Vis. Res..

[24] Akopian A., Kumar S., Ramakrishnan H., Roy K., Viswanathan S., Bloomfield S.A. (2017). Targeting neuronal gap junctions in mouse retina offers neuroprotection in glaucoma. J. Clin. Investig..

[25] Akopian A., Kumar S., Ramakrishnan H., Viswanathan S., Bloomfield S.A. (2019). Amacrine cells coupled to ganglion cells via gap junctions are highly vulnerable in glaucomatous mouse retinas. J. Comp. Neurol..

[26] Della Santina L., Inman D.M., Lupien C.B., Horner P.J., Wong R.O.L. (2013). Differential Progression of Structural and Functional Alterations in Distinct Retinal Ganglion Cell Types in a Mouse Model of Glaucoma. J. Neurosci..

[27] Weitlauf C., Ward N.J., Lambert W.S., Sidorova T.N., Ho K.W., Sappington R.M., Calkins D.J. (2014). Short-term increases in transient receptor potential vanilloid-1 mediate stress-induced enhancement of neuronal excitation. J. Neurosci..

[28] Ward N.J., Ho K.W., Lambert W.S., Weitlauf C., Calkins D.J. (2014). Absence of Transient Receptor Potential Vanilloid-1 Accelerates Stress-Induced Axonopathy in the Optic Projection. J. Neurosci..

[29] Morquette B., Morquette P., Agostinone J., Feinstein E., McKinney R.A., Kolta A., Di Polo A. (2015). REDD2-mediated inhibition of mTOR promotes dendrite retraction induced by axonal injury. Cell Death Differ..

[30] Feng L., Zhao Y., Yoshida M., Chen H., Yang J.F., Kim T.S., Cang J., Troy J.B., Liu X. (2013). Sustained Ocular Hypertension Induces Dendritic Degeneration of Mouse Retinal Ganglion Cells That Depends on Cell Type and Location. Investig. Opthalmol. Vis. Sci..

[31] Tran N.M., Shekhar K., Whitney I.E., Jacobi A., Benhar I., Hong G., Yan W., Adiconis X., Arnold M.E., Lee J.M. (2019). Single-Cell Profiles of Retinal Ganglion Cells Differing in Resilience to Injury Reveal Neuroprotective Genes. Neuron.

[32] Agostinone J., Alarcon-Martinez L., Gamlin C., Yu W.-Q., Wong R.O.L., Di Polo A. (2018). Insulin signalling promotes dendrite and synapse regeneration and restores circuit function after axonal injury. Brain.

[33] Kalesnykas G., Oglesby E.N., Zack D.J., Cone F.E., Steinhart M.R., Tian J., Pease M.E., Quigley H.A. (2012). Retinal Ganglion Cell Morphology after Optic Nerve Crush and Experimental Glaucoma. Investig. Opthalmol. Vis. Sci..

[34] Leung C.K., Weinreb R.N., Li Z.W., Liu S., Lindsey J.D., Choi N., Liu L., Cheung C.Y., Ye C., Qiu K. (2011). Long-Term In Vivo Imaging and Measurement of Dendritic Shrinkage of Retinal Ganglion Cells. Investig. Opthalmol. Vis. Sci..

[35] Mak H.K., Ng S.H., Ren T., Ye C., Leung C.K. (2020). Impact of PTEN/SOCS3 deletion on amelioration of dendritic shrinkage of retinal ganglion cells after optic nerve injury. Exp. Eye Res..

[36] Jakobs T.C., Libby R.T., Ben Y., John S.W.M., Masland R.H. (2005). Retinal ganglion cell degeneration is topological but not cell type specific in DBA/2J mice. J. Cell Biol..

[37] El-Danaf R.N., Huberman A.D. (2015). Characteristic Patterns of Dendritic Remodeling in Early-Stage Glaucoma: Evidence from Genetically Identified Retinal Ganglion Cell Types. J. Neurosci..

[38] Bhandari A., Smith J.C., Zhang Y., Jensen A.A., Reid L., Goeser T., Fan S., Ghate D., Van Hook M.J. (2019). Early-Stage Ocular Hypertension Alters Retinal Ganglion Cell Synaptic Transmission in the Visual Thalamus. Front. Cell. Neurosci..

[39] Chou T.-H., Kocaoglu O.P., Borja D., Ruggeri M., Uhlhorn S.R., Manns F., Porciatti V. (2011). Postnatal Elongation of Eye Size in DBA/2J Mice Compared with C57BL/6J Mice: In Vivo Analysis with Whole-Eye OCT. Investig. Opthalmol. Vis. Sci..

[40] Nadal-Nicolás F.M., Salinas-Navarro M., Vidal-Sanz M., Agudo-Barriuso M. (2015). Two methods to trace retinal ganglion cells with fluorogold: From the intact optic nerve or by stereotactic injection into the optic tract. Exp. Eye Res..

[41] Sergeys J., Etienne I., Van Hove I., Lefevere E., Stalmans I., Feyen J.H.M., Moons L., Van Bergen T. (2019). Longitudinal In Vivo Characterization of the Streptozotocin-Induced Diabetic Mouse Model: Focus on Early Inner Retinal Responses. Investig. Opthalmol. Vis. Sci..

[42] Crish S.D., Sappington R.M., Inman D.M., Horner P.J., Calkins D.J. (2010). Distal axonopathy with structural persistence in glaucomatous neurodegeneration. Proc. Natl. Acad. Sci. USA.

[43] Schindelin J., Arganda-Carreras I., Frise E., Kaynig V., Longair M., Pietzsch T., Preibisch S., Rueden C., Saalfeld S., Schmid B. (2012). Fiji: An open-source platform for biological-image analysis. Nat. Methods.

[44] Reinhard K., Li C., Do Q., Burke E.G., Heynderickx S., Farrow K. (2019). A projection specific logic to sampling visual inputs in mouse superior colliculus. eLife.

[45] Cuntz H., Forstner F., Borst A., Häusser M. (2010). One Rule to Grow Them All: A General Theory of Neuronal Branching and Its Practical Application. PLoS Comput. Biol..

[46] R Core Team (2020). R: A Language and Environment for Statistical Computing.

[47] Ho J., Tumkaya T., Aryal S., Choi H., Claridge-Chang A. (2019). Moving beyond P values: Data analysis with estimation graphics. Nat. Methods.

[48] Nuzzi R., Tridico F. (2017). Glaucoma: Biological Trabecular and Neuroretinal Pathology with Perspectives of Therapy Innovation and Preventive Diagnosis. Front. Neurosci..

