# Supplementary material for: A Fair Assessment of Evaluation Tools for the Murine Microbead Occlusion Model of Glaucoma
Source: Int J Mol Sci. 2021 May 26;22(11):5633. doi: 10.3390/ijms22115633 (PMC8199180; doi:10.3390/ijms22115633)
Supplement: Supplementary file 1 [file ijms-22-05633-s001.zip › ijms-1212090-supplementary.pdf]

International Journal of *Molecular Sciences*

MDPI

Article

A Fair Assessment of Evaluation Tools for the Murine Microbead Occlusion Model of Glaucoma

Marie Claes [1], Joana R. F. Santos [1,2,3,4], Luca Masin [1], Lien Cools [1], Benjamin M. Davis [5,6], Lut Arckens [1], Karl Farrow [1,2,3,4], Lies De Groef [1] and Lieve Moons [1,*]

1 Department of Biology & Leuven Brain Institute, KU Leuven, 3000 Leuven, Belgium; marie.claes@kuleuven.be (M.C.); Joana.Santos@nerf.be (J.R.F.S.); luca.masin@kuleuven.be (L.M.); lien.cools@kuleuven.be (L.C.); lut.arckens@kuleuven.be (L.A.); Karl.Farrow@nerf.be (K.F.); lies.degroef@kuleuven.be (L.D.G.)

2 Neuro-Electronics Research Flanders, 3001 Leuven, Belgium

3 Vlaams Instituut voor Biotechnologie (VIB), Center for Brain & Disease Research, 3000 Leuven, Belgium

4 Imec, 3001 Leuven, Belgium

5 Institute of Ophthalmology, University College London, London EC1V 9EL, UK; benjamin.davis@stfc.ac.uk

6 Central Laser Facility, Science and Technologies Facilities Council, UK Research and Innovation, Didcot OX11 0QX, UK

* Correspondence: lieve.moons@kuleuven.be

Supplementary Table

Table S1. Effect size statistics for electroretinogram (ERG) parameters.

Parameter	Hedges'g	$CI_{95\%}$
a-wave amplitude	0.54	[−0.58 − 1.46]
a-wave latency	−0.52	[−1.44 − 0.50]
b-wave amplitude	0.06	[−0.95 − 1.14]
b-wave latency	−0.71	[−1.63 − 0.52]
OP1 amplitude	−0.75	[−1.67 − 0.38]
OP1 latency	0.59	[−0.52 − 1.64]
OP2 amplitude	−0.68	[−1.50 − 0.45]
OP2 latency	0.40	[−0.75 − 1.67]
OP3 amplitude	−0.09	[−1.04 − 1.01]
OP3 latency	−0.15	[−1.10 − 0.93]

Supplementary Figures

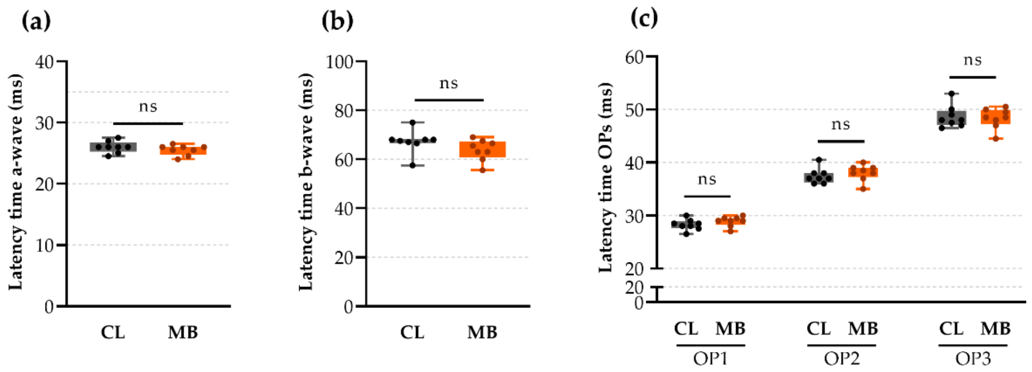

Figure S1. Latency time of full-field flash electroretinogram (ERG) recordings at five weeks post microbead injection. (**a**–**c**) Bar graphs showing the unaltered latency time of the a-wave (**a**) , b-wave (**b**) and oscillatory potentials (OPs) (**c**) components. Unpaired two-tailed *t*-tests (**a**,**b**) or one-way ANOVA test with Tukey's post-hoc tests (**c**), ns = non-significant. CL = contralateral eyes, MB = microbead-injected eyes.

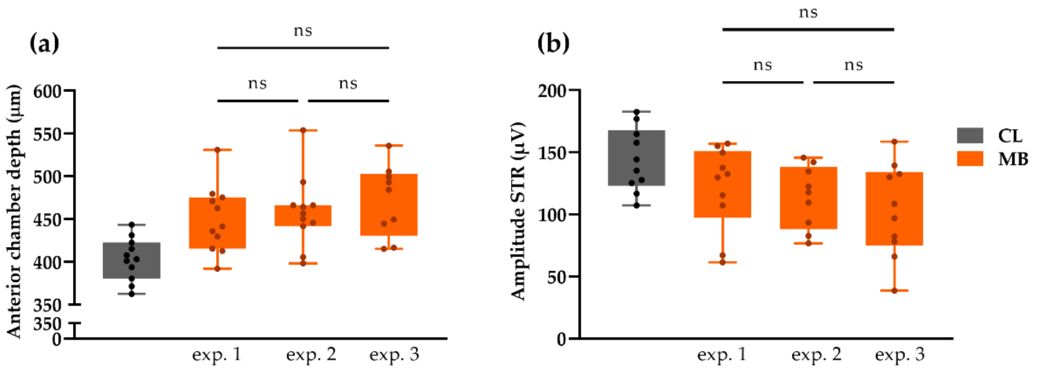

Figure S2. Experiment-to-experiment variability and reproducibility of two evaluation tools for success rate after microbead injection. (**a**,**b**) The variation in anterior chamber depth and scotopic threshold response (STR) amplitude values, as well as the mean enlargement and decrease, respectively, upon microbead injection, are shown to be equivalent in three different independent experiments. One-way ANOVA with Tukey's post-hoc test, ns = non-significant. Key: CL = contralateral eyes, MB = microbead-injected eyes and STR = positive scotopic threshold response.
